# Aortoenteric Fistula: A Rare Cause of Gastrointestinal Bleeding

**DOI:** 10.7759/cureus.82361

**Published:** 2025-04-16

**Authors:** Frederica H Ferreira, Inês Ambrioso, Filipe Rodrigues, Pedro A Martins, Graça Amaro

**Affiliations:** 1 Internal Medicine, District Hospital of Santarém, Santarém, PRT; 2 Vascular Surgery, District Hospital of Santarém, Santarém, PRT

**Keywords:** aortic abdominal aneurysm, aortoenteric fistula, endovascular aneurysm repair, gastro intestinal bleeding, herald bleeding

## Abstract

A rare cause of gastrointestinal bleeding is an aortoenteric fistula, which typically occurs after previous aortic surgeries and is referred to as a secondary aortoenteric fistula (SAEF).

A 74-year-old male smoker with a medical history of open surgical repair of an infrarenal aortic aneurysm in 2009 was admitted to the emergency department with shortness of breath on mild exertion, weakness, and melena for the past two weeks. During hospitalization, he experienced a sudden episode of abdominal pain, abundant hematochezia, and shock. An abdominal computed tomography (CT) angiography revealed a distal aortic anastomotic pseudoaneurysm with contrast extravasation, confirming the presence of an aortoenteric fistula. The patient underwent endovascular abdominal aortic aneurysm repair (EVAR) via bilateral femoral access, and empirical antibiotic therapy was initiated.

Aortoenteric fistulas should be considered in patients with a history of aortic aneurysm repair who present with recurrent melena or hematochezia, despite normal upper and lower endoscopic examinations. A herald bleed, occurring prior to a massive hemorrhage, is crucial for early diagnosis, as there may be limited time for further investigations.

## Introduction

Gastrointestinal bleeding is one of the most common gastrointestinal conditions requiring medical attention. It can range from mild to severe and may be life-threatening. Its incidence increases with age, with older patients being more frequently affected [1].

Non-variceal etiologies account for 80-90% of upper gastrointestinal bleeding, with peptic ulcer disease being the most common cause. Vascular lesions, tumors, Mallory-Weiss syndrome, and esophagitis are also potential causes of gastrointestinal bleeding [2].

A potentially fatal and rare cause of bleeding is a fistula between the aorta and the gastrointestinal tract, commonly referred to as an aortoenteric fistula. It typically occurs after previous aortic surgeries and is known as a secondary aortoenteric fistula (SAEF) [3].

SAEFs involving the ileal segment of the small intestine are rarely reported following endovascular aortic repair. Though SAEFs are an uncommon complication, they are potentially life-threatening after aortic aneurysm reconstruction. Despite the clinical importance of identifying the underlying mechanism of fistula formation, the exact etiology remains unclear. It has been proposed that chronic graft infection or mechanical factors, such as aortic pulsation pressure, may contribute to their development. However, these conclusions are often based solely on clinical presentation or intraoperative findings.

To date, only 25 case reports of SAEFs have been published. Among these, just six address the potential mechanisms of fistula formation and include pathological findings, and only two provide detailed pathological images. As such, the precise pathophysiological mechanism behind SAEF formation has yet to be clearly defined through pathological analysis [4].

Here, we present a case of gastrointestinal bleeding secondary to an aorto-ileal fistula in a patient who had undergone open surgical repair of an abdominal aortic aneurysm (AAA) 12 years prior to this admission.

## Case presentation

A 74-year-old male smoker, with a medical history of ischemic heart disease, aortic valve stenosis, heart failure with reduced ejection fraction, atrial fibrillation on anticoagulation therapy, and a previous open surgical repair of an infrarenal aortic aneurysm in 2009, was admitted to the emergency department with shortness of breath on mild exertion, weakness, and melena for the past two weeks. Physical examination revealed pale, discolored mucous membranes, with a blood pressure of 93/37 mmHg and a heart rate of 75 bpm. Hemoglobin was 5.9 g/dL, and a blood transfusion with two units of red blood cells was performed, resulting in hemodynamic stabilization. Anticoagulation therapy was discontinued, the patient was placed on nil by mouth, and esomeprazole infusion was started due to suspicion of upper gastrointestinal bleeding. The patient was subsequently admitted for hospitalization.

Endoscopic studies, including both upper and lower endoscopy, were performed but yielded no significant findings. An abdominal and pelvic computed tomography (CT), unfortunately performed without intravenous (IV) contrast due to the patient's elevated serum creatinine levels, revealed a saccular aneurysm above the aortic bifurcation, along with intraperitoneal, perisplenic, and perihepatic fluid.

During hospitalization, episodes of melena were recurrent, sometimes accompanied by hematochezia. Frequent blood transfusions were administered to maintain the patient’s hemodynamic stability and keep hemoglobin levels above 7 g/dL.

**Figure 1:**
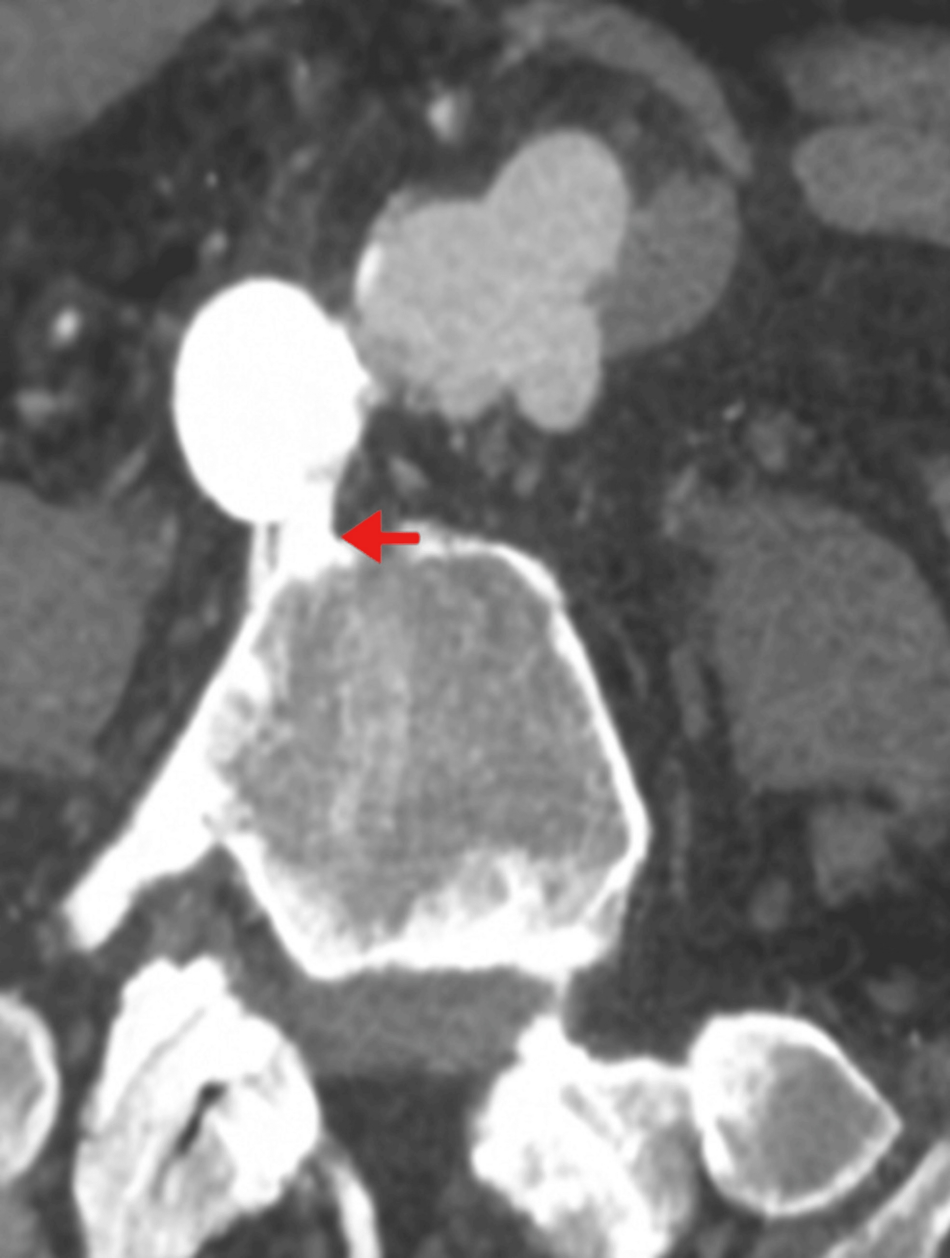
Abdominal CT angiography showing an aortic distal anastomotic pseudo-aneurysm with an aortoenteric fistula (red arrow).

A sudden episode of abdominal pain, accompanied by abundant hematochezia and shock, occurred. An abdominal CT angiography was performed, revealing a distal aortic anastomotic pseudoaneurysm measuring approximately 3 x 2 cm, with bubbles inside, indicating an aortoenteric fistula (Figure 1).

**Figure 2:**
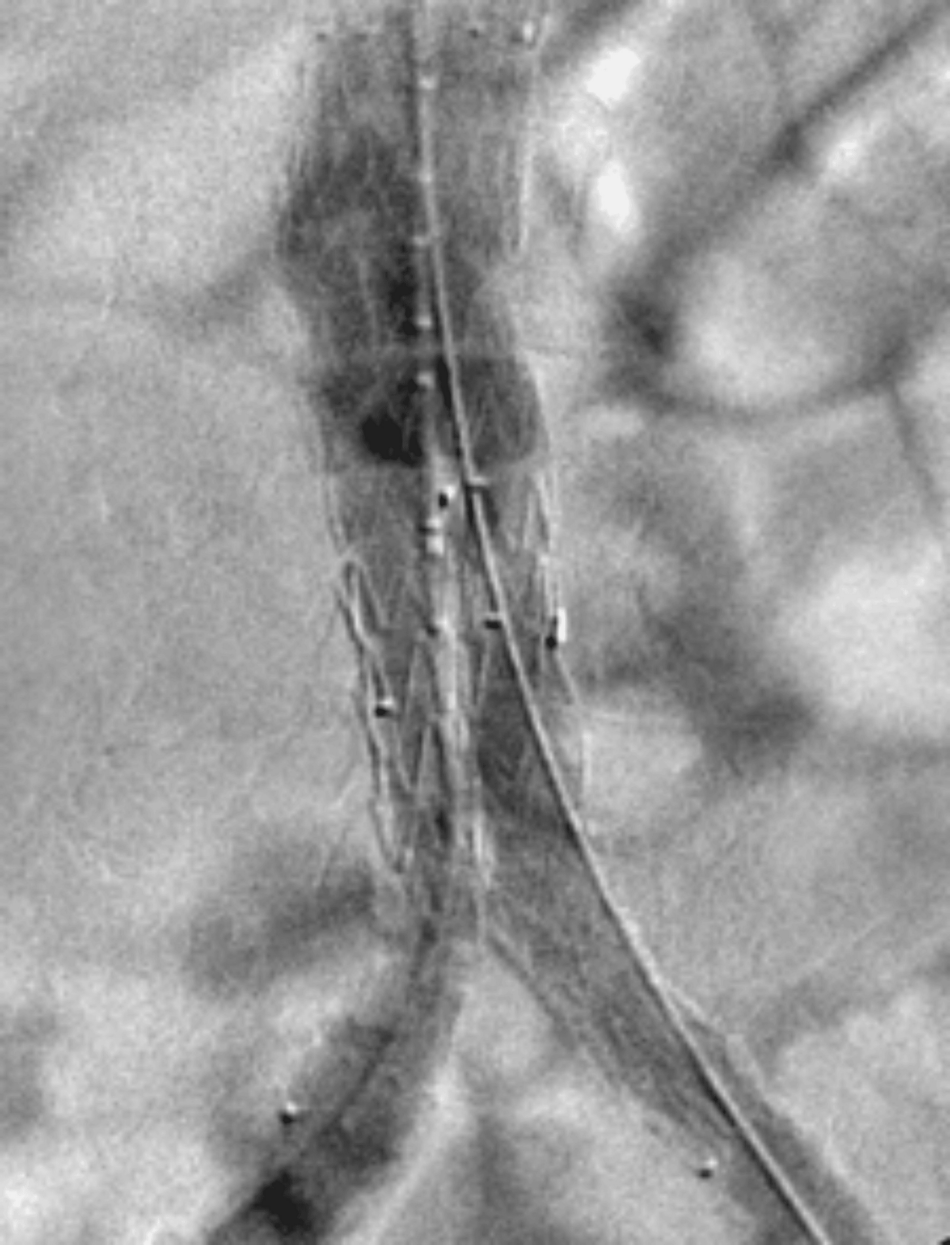
Under local anesthesia and sedation, the EVAR technique was performed via bilateral femoral access. EVAR: endovascular abdominal aortic aneurysm repair

The patient was transferred to the intensive care unit (ICU) for hemodynamic support. Emergency vascular surgery was scheduled, and the endovascular AAA repair (EVAR) technique was performed via bilateral femoral access. A bifurcated infrarenal aortic endoprosthesis with bilateral common iliac extensions (Zenith Alpha, Cook Medical, USA) was implanted (Figure 2). After the procedure, the patient remained in the ICU for a few days before being transferred to the hospital ward.

**Figure 3:**
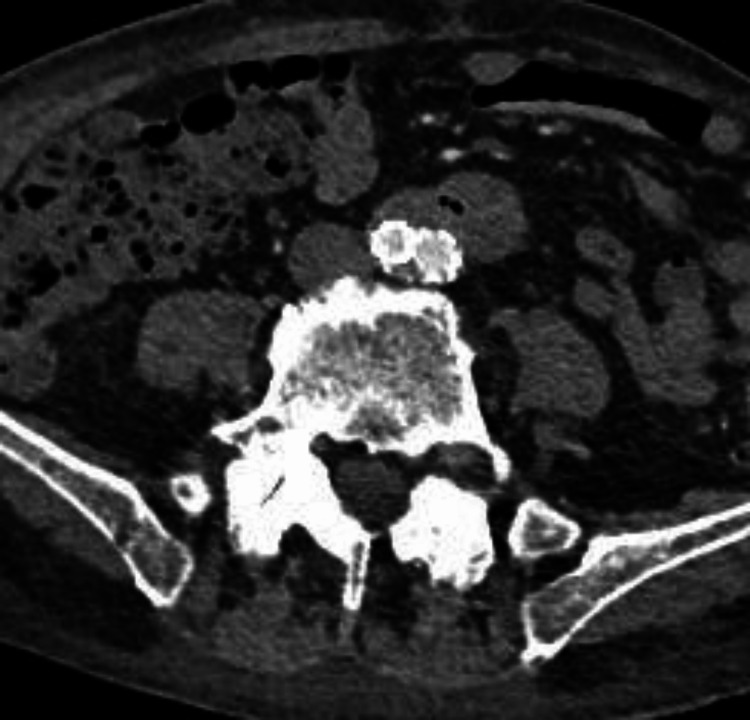
Postoperative follow-up with abdominal CT angiography showed no complications in the intervention site.

The patient's postoperative recovery progressed favorably with a light diet and empirical antibiotic therapy. Meropenem 1 g was administered every eight hours, and there was no recurrence of gastrointestinal bleeding. Postoperative follow-up with abdominal CT angiography showed no complications in the intervention site (Figure 3). Long-term antibiotic therapy with levofloxacin 500 mg a day was continued for six months after discharge. No signs of prosthesis infection were observed. Unfortunately, a diagnosis of large cell adenocarcinoma was made through a pulmonary nodule biopsy, which ultimately resulted in the patient's death six months later.

## Discussion

Aortoenteric fistulas were first described in 1829 by Sir Astley Cooper [2].

Aortoenteric fistulas can be classified as primary and secondary. Primary aortoenteric fistulas (PAEFs) typically represent a spontaneous communication between the lumen of an aortic aneurysm and an intestinal loop, occurring without prior grafting. In contrast, SAEFs develop following aortic reconstructive surgery or endovascular repair [2]. Two hypotheses have been proposed regarding the origin of SAEFs: (a) One hypothesis suggests that continuous aortic pulsation exerts mechanical stimulation on the walls of the intestinal tract and adjacent arteries. This theory is supported by the fact that most SAEFs involve the third or fourth portion of the duodenum, which is compressed between the superior mesenteric artery and the abdominal aorta in the retroperitoneal space. (b) The second hypothesis posits that SAEFs may result from a local inflammatory response due to prosthetic infection during the initial surgery. This is supported by the detection of bacterial species, such as *Staphylococcus*, which are not typically found in the intestine, in aortic prostheses in cases of SAEF [4].

PAEFs were the most commonly encountered before 1960. However, recently, the incidence of SAEF has increased, now accounting for 4% of aortic repair and reconstruction complications. This rise is attributed to the growing number of revascularizations for aortic aneurysms and severe aortoiliac atherosclerosis [5]. Aortoenteric fistulas most commonly involve the third and fourth portions of the duodenum (88%), with less frequent involvement of the more proximal duodenum (8%), jejunum, or ileum [6,7]. Aortoenteric fistula is more common in males, with a male-to-female ratio of 8:1 for SAEF, reflecting the incidence of AAA and aortic surgery [8].

Clinical suspicion is essential for prompt diagnosis. A classical triad with gastrointestinal bleeding, abdominal pain, and a pulsatile mass has been suggested for diagnosing aortoenteric fistulas. However, this presentation is rare, making diagnosis highly challenging [2,5,8].

Bleeding episodes can range from mild to massive, life-threatening hemorrhages. A self-limited bleeding episode before a massive, life-threatening bleed, known as a herald bleed, may occur in approximately 20-75% of cases, as seen in our patient. However, massive bleeding can occur hours or even months later. Retrospective studies have shown that some patients present initially with massive bleeding and hemorrhagic shock, while others experience recurrent melena or hematochezia over one to three months. When a massive hemorrhage occurs a few hours after the initial bleed, a window for early diagnosis is created, although a high level of clinical suspicion is necessary, and the patient's medical history becomes critical for diagnosis [5-9]. Abdominal pain is reported in 35% of patients. Interestingly, our patient experienced moderate, generalized abdominal pain, dull in nature, with a soft and depressible abdomen, despite exhibiting generalized tenderness on deep palpation. This suggests that abdominal pain should be regarded as a red flag and addressed with appropriate seriousness by clinicians [8].

Early diagnosis and intervention have a preventive effect on mortality and morbidity [9].

Aortoenteric fistula is challenging to diagnose before surgery, with preoperative diagnosis rates ranging from only 14.3% to 36% [8].

CT angiography has become the most commonly used procedure to confirm the diagnosis, with a diagnostic sensitivity of 40-90% and specificity ranging from 33% to 100%. Findings suggesting the presence of a fistula include bubbles around the aorta, the absence of a fat plane between the aorta and digestive tract, edema of the intestinal wall surrounding the aorta, and extravasation of contrast medium from the aorta into the intestinal lumen. While aortic contrast extravasation into the bowel lumen is uncommon, it is the most distinctive characteristic of an aortoenteric fistula [2,8]. In our case, bubbles were observed.

Endoscopic studies are the initial method of choice for assessing gastrointestinal bleeding, but their accuracy for diagnosing aortoenteric fistula is only 25-40%. A retrospective study showed that CT angiography can identify a fistula in 35% of cases, while endoscopic methods can identify only 25%. This discrepancy is due to the difficulty in reaching the third portion of the duodenum or, in cases of massive bleeding, locating the origin of the bleed [8].

The treatment of choice for aortoenteric fistula is surgical repair. Conventional surgery, involving explantation and in situ reconstruction, remains the gold standard for definitive treatment. However, it is associated with high morbidity and mortality [8].

There are several surgical options to restore arterial perfusion and intestinal continuity, with no significant differences in the methods used to repair PAEF and SAEF. These options primarily fall into two categories: in situ reconstructions and extra-anatomic bypass with aortic ligation, although both have been associated with less favorable outcomes [8].

Minimally invasive techniques, such as EVAR, have been tried and show short-term success. EVAR is typically recommended for patients who are not candidates for open surgery due to poor general health or hemodynamic instability. Because it offers better control of aortic bleeding, EVAR is often used as a bridging measure until definitive open surgery can be performed. However, EVAR is associated with a significant early-to-midterm infection rate, leaving patients at considerable risk of infection. Therefore, these techniques should be accompanied by intensive antibiotic therapy for infection control [5,10-12].

## Conclusions

Aortoenteric fistulas are life-threatening, and delayed diagnosis can lead to catastrophic bleeding and mortality. In patients with hemorrhage, especially those with recurrent melena or hematochezia despite normal upper and lower endoscopic examinations, suspicion of aortoenteric fistula should be considered. A thorough review of the medical history is essential, particularly in patients with gastrointestinal bleeding and a history of aortic aneurysm repair. Clinical suspicion in patients with herald bleeding before a massive bleed is crucial for early diagnosis, as there may be limited time for further investigations.

Fortunately, with advances in surgical techniques, EVAR should be considered a viable definitive treatment for frail patients with limited life expectancy, as demonstrated by the authors in this case. Although this is a vascular surgery case, general surgeons also play a crucial role in selecting the appropriate method for restoring the intestinal tract, a decision that appears to be significantly linked to subsequent morbidity and mortality.
